# Relationship between Aurora-A V57I Polymorphism and the Risk of Cancer: A Meta-Analysis and Trial Sequential Analysis

**DOI:** 10.7150/jca.40567

**Published:** 2020-03-05

**Authors:** Guangyuan Chen, Cong Hu, Yuxuan Song, Mengxi Xiu, Yiling Zhang, Penghui Lai, Yunyan Li, Xiaoqiang Liu, Peng Huang

**Affiliations:** 1 The Second Clinical Medical School, Nanchang University, Nanchang, Jiangxi 330006, China.; 2 Department of Urology, Tianjin Medical University General Hospital, Tianjin 300052, China.; 3 Center for Evidence-based Medicine, School of Public Health, Nanchang University, Nanchang 330006, China.; 4 Jiangxi Province Key Laboratory of Preventive Medicine, School of Public Health, Nanchang University, Nanchang 330006, China.

**Keywords:** Aurora-A, polymorphism, meta-analysis, cancer

## Abstract

**Background**: It is still conflicting for the correlation between cancer susceptibility and Aurora-A V57I (rs1047972) gene variant from the published researches. This meta-analysis was performed to access the correlation between cancer susceptibility and Aurora-A rs1047972 gene polymorphism by using meta-analysis methods.

**Methods**: Eligible studies published before Nov 1, 2019 were systematically searched in PMC, PubMed, EMBASE, Web of Science, Cochrane Library Database, China National Knowledge Infrastructure, Wanfang databases, in order to collect qualified case-control or cohort studies. The odds ratio (OR) and its 95% confidence interval (95%CI) were used to evaluate the correlation between Aurora-A rs1047972 gene polymorphism and cancer risk. Sensitivity analysis was used to examine the stability of the results; Egger's test and Begg's funnel chart were used to assess possible publication bias. Trial sequential analysis (TSA) was used to access whether the sample size of our meta-analysis was sufficient.

**Results**: The sample set extracted from 24 case-control studies involving 35,926 subjects (14,639 cases and 21,287 controls) for the association of Aurora-A rs1047972 gene polymorphism with cancer susceptibility. In our meta-analysis, Aurora-A rs1047972 polymorphism was associated with an increased risk of cancer susceptibility in overall populations (GA+GG vs. AA: P=0.039, OR=1.106; 95% CI 1.005-1.218; AA vs. GG: P=0.003, OR= 0.814; 95% CI, 0.710-0.934), and the GA/GG variant might be a risk factor for cancer susceptibility. In the stratified analysis by ethnicity, we found a significant association between Aurora-A rs1047972 variant and the susceptibility of the cancer in Caucasian population. In a subgroup analysis by cancer type, we observed a significantly increased susceptibility of lung cancer. In addition, an increased risk was found between Aurora-A rs1047972 polymorphism and cancer susceptibility in MALDI-TOF group and among population-based study (PB) patients. Our results were in a sufficiently large number of participants according to TSA and did not require more studies to confirm such association.

**Conclusion**: Our meta-analysis revealed that the susceptibility of cancer was associated with Aurora-A rs1047972 polymorphism, especially in Caucasians. And the GA/GG variant might be a risk factor for cancer susceptibility.

## Introduction

Aurora Kinase A (AURKA) is a serine/threonine kinase, which belongs to the Aurora kinase family 1. In the process of mitosis, AURKA plays an important role in the cell cycle by participating in the separation and maturation of the centrosome and the establishment of the bipolar mitotic spindles, ensuring the correct separation of chromosomes and the completion of cytoplasmic division 2. Abnormal amplification and high expression of AURKA are common in a variety of gastric adenocarcinomas and esophageal adenocarcinomas 3. Recent studies have suggested that AURKA can participate in some important cell signaling pathways such as the Wnt signaling pathway. It directly or indirectly regulates the expression of some important proteins, and has extraordinary impact on the development of tumors 4-5.

The AURKA gene owns a lot of polymorphisms, so far, at least 6 AURKA polymorphism sites have been found, of which the most investigated were two common non-synonymous single nucleotide polymorphisms (SNPs) (F31I and V57I). However, there were at least four meta-analyses indicating F31I SNP was a low protective factor in the development of tumors. So we only analyzed the SNP V57I and did not analyze SNP F31I, as we needed to be sure that there was no redundancy that may be caused out of the publication of the research for SNP F31I. Gene polymorphism has been reported to be a significant factor and it might increase the susceptibility of cancer 6-7. At present, more and more epidemiologic researches are centered on correlation between rs1047972 (V57I) gene polymorphism and cancers risk, but the conclusions are still controversial. Based on it, this meta-analysis was performed to investigate whether the Aurora-A rs1047972 (V57I) gene polymorphism was correlated with the susceptibility of cancer by widely collecting the reported investigations.

## Materials and Methods

### Literature retrieval

Eligible studies were systematically searched in PMC, Cochrane Library database, PubMed, EMBASE, Web of Science, China National Knowledge Infrastructure, Wanfang databases. And we also performed manual search to find relevant studies. English search strategy: (Aurora-A OR BTAK OR STK15 OR AIKI OR rs1047972) AND (cancer OR neoplasm OR tumor OR carcinoma) AND (SNP or variant or genotype or polymorphism). We searched the studies published before Nov 1, 2019, and also searched relevant degree papers.

### Inclusion and exclusion criteria

#### Inclusion criteria

(1) Case-control or cohort studies on the association between Aurora-A rs1047972 gene polymorphism and cancers risk; (2) The genotypes of the study followed with Hardy-Weinberg equilibrium (HWE); (3) Alleles or genotypes frequencies in control groups and case groups could be received from the studies; (4) English and Chinese literature.

#### Exclusion criteria

(1) Unable to extract useful data or results; (2) The Aurora-A rs1047972 polymorphism was not included or the results were not about cancers susceptibility; (3) The studies published repeatedly and the one included the largest sample size.

### Data extraction and literature quality evaluation

Using the Newcastle-Ottawa Scale (the NOS scale) to evaluate the included articles, if the score of the articles were more than or equal to 5 points, the quality of the articles was considered to be high. Two authors independently read the retrieved article titles and abstracts. After excluding the articles that did not meet the inclusion criteria, the full review of the retrieved articles probably meeting the inclusion criteria was performed to determine its qualifications for final incorporation after cross-checking by the two research evaluators. The extraction information mainly includes: the name of the first author of the studies, ethnicity, original country, the year of publication, sample size, source of controls (population-based controls and hospital-based controls), genotyping methods, the number of cases and controls for Aurora-A rs1047972 genotypes and so on.

### Statistical analyses

We performed meta-analysis using STATA 12.0 software, and the count data was expressed using odds ratio (OR) and its 95% confidence interval (95% CI). The heterogeneity between the results of each included study was tested by chi-square test. When there was a small statistical homogeneity between these studies (P>0.1 and* I^2^*<50%), a meta-analysis was performed by using a fixed-effect model; if there was a huge statistical heterogeneity between these studies (P<0.1 or *I^2^*> 50%), we analyzed the results using a random-effect model, if necessary, a subgroup analysis of related factors that may lead to heterogeneity will be performed. The pooled ORs were performed for these models (1) AA vs.GG, (2) GA vs. AA, (3) AA+GA vs.GG and (4) GG+GA vs. AA, respectively. We use Egger's test and Begg's funnel chart to evaluate the publication of bias. Each time a document was removed for sensitivity analysis. Subgroup analysis was carried out, as it needed.

### Trial Sequential Analysis (TSA)

We used the TSA v0.9.5.10 Beta software to perform the trial sequential analysis. Our study sets the odds ratio reduction to 20%, the first type of error α=0.05, and the second type of error β=0.2 to evaluate the required information size (RIS). At the same time, if the cumulative Z value crosses the RIS threshold, the results are considered statistically significant. Therefore, it can be considered the sample size of the accumulated evidence is sufficient. However, if the cumulative Z value does not cross the RIS threshold, it means the sample size is not sufficient. And it still needs more studies to confirm the results.

## Results

### Characteristics of the selected studies

**Figure 1:**
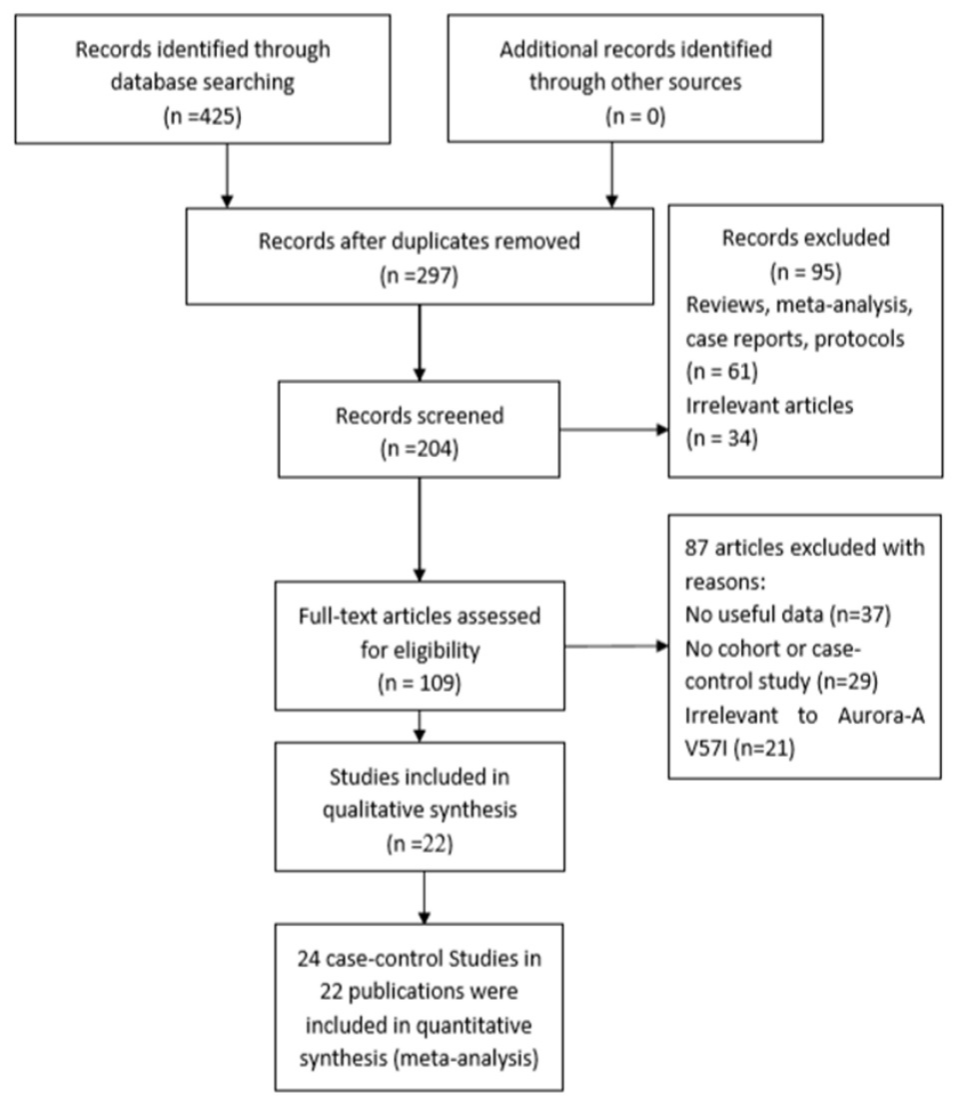
Flowchart illustrating the search strategy for Aurora-A V57I variant and the risk of cancer.

The search strategy resulted in a total of 425 potentially relevant articles (Figure 1). The characteristics of the included studies were listed in Table 1. For Aurora-A V57I (rs1047972) polymorphism, 22 articles 8-29 were investigated. However, there were three independent groups in Dicioccio's study 14, and we treated them separately. Finally, 24 case-control studies were included in the present meta-analysis. Of these 24 studies (including 14,639 cases and 21,287 controls), seven studied the association between Aurora-A rs1047972 variant and the risk of breast cancer, three studied the association between Aurora-A rs1047972 variant and the susceptibility of ovarian cancer and three studied lung cancer. The other studied the susceptibility of bladder, cervical, gastric and colorectal cancer. As far as the genotyping methods, 8 were using PCR, 11 were using TaqMan, 4 were using PCR-RFLP, and one was using MALDI-TOF. There are 10 studies for the Asian populations and 14 studies for the Caucasian populations. The NOS scores of the 24 documents were all more than 5 it means that all of them were high quality studies (Table 1; Figure 1).

### Meta-analysis results

#### Association between the risk of cancer and Aurora-A rs1047972 polymorphism in the total population

**Figure 2:**
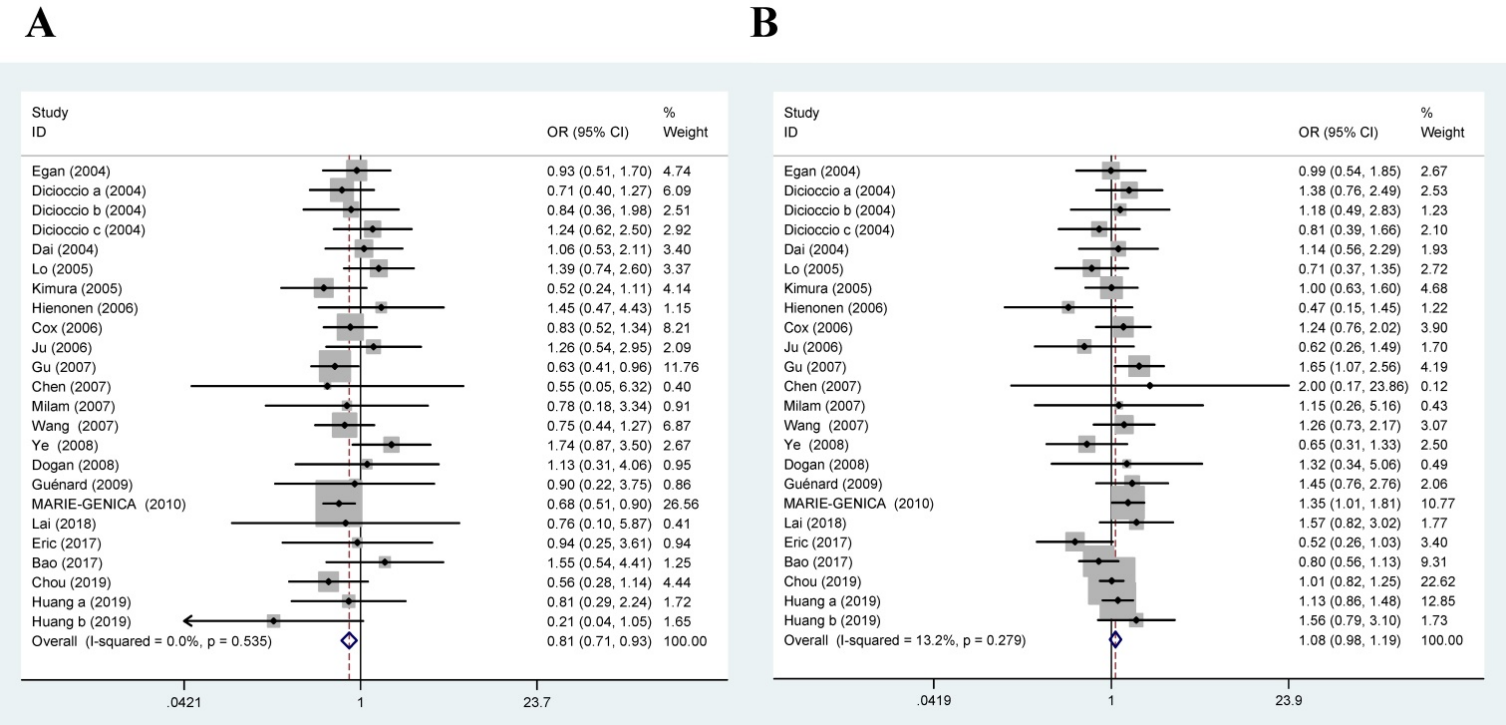

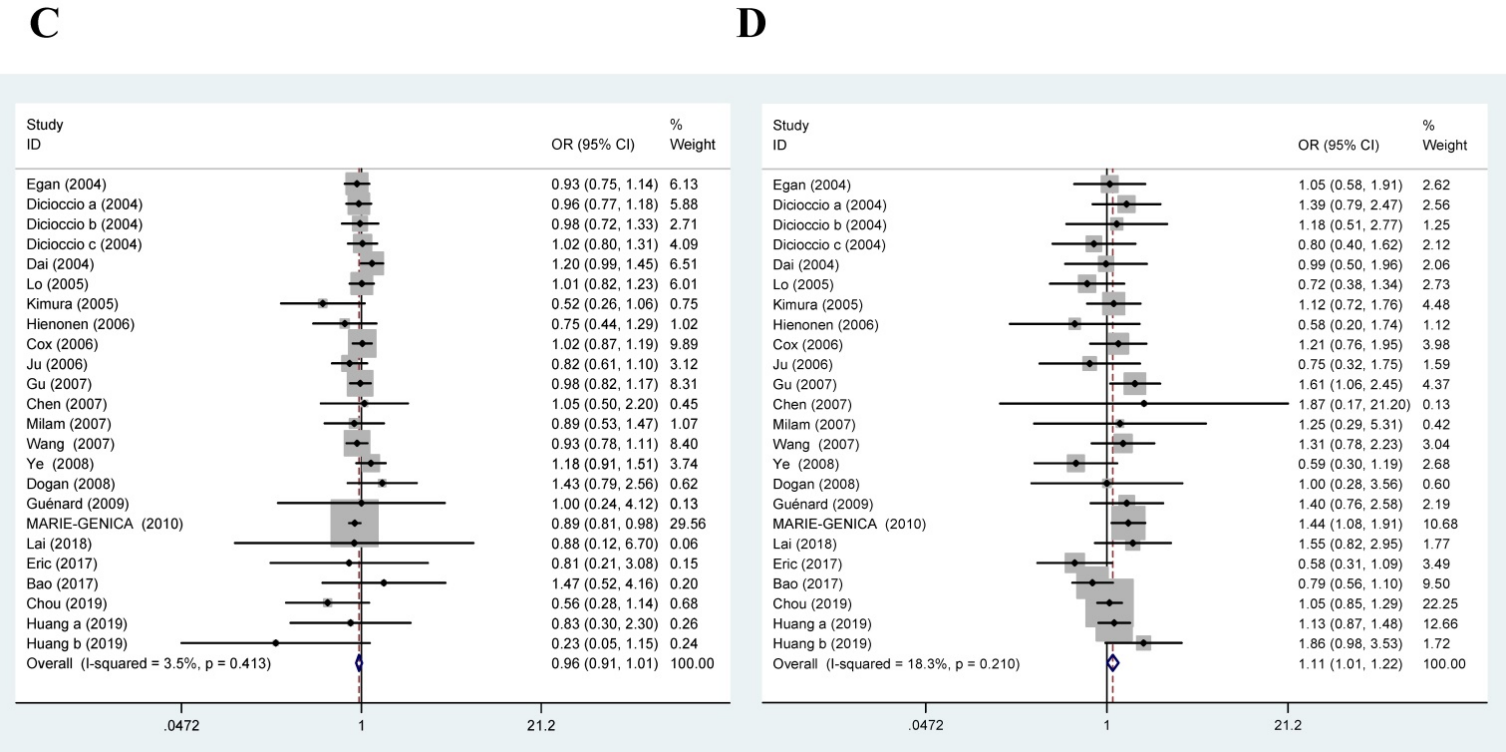
Forest plot of association between Aurora-A V57I variant and cancer (A: AA vs.GG; B: GA vs. AA; C: AA+GA vs.GG; D: GG+GA vs. AA).

24 case-control studies including 14,639 cancer cases and 21,287 normal controls were investigated. As illustrated in Table 2, a significant increased risk was observed for Aurora-A rs1047972 gene polymorphism and cancer susceptibility in overall population (GA+GG vs. AA: P=0.039, OR=1.106, 95% CI 1.005-1.218; AA vs. GG: P=0.003, OR=0.814, 95% CI 0.710-0.934). And the AA genotype carriers have a slightly lower incidence of cancer compared to that of GG or GA carriers and the GG or GA variant might be a risk factor for cancer susceptibility in overall populations (Table 2; Figure 2).

#### Association between cancer risk and Aurora-A rs1047972 polymorphism in subgroup analysis by ethnicity

Subgroup analysis of different ethnicities was carried out. We found a significant association between Aurora-A rs1047972 variant and the susceptibility of the cancer in Caucasian population (AA vs. GG: P=0.005, OR=0.797, 95% CI 0.681-0.933; GA vs. AA: P=0.009, OR=1.234, 95% CI 1.054-1.445; GA+GG vs. AA: P=0.004, OR=1.254, 95% CI 1.077-1.461). Our results indicated that GA/GG genotype carriers have a higher risk of cancer compared to AA carriers and the AA variant might be a protective factor for cancer susceptibility in Caucasian population. However, we did not find any association between Aurora-A rs1047972 variant and cancer risk in Asian population (Table 2).

#### Association between Aurora-A rs1047972 polymorphism and cancer risk in subgroup analysis by cancer type

In a stratified analysis by different types of cancer, our results suggested that Aurora-A rs1047972 variant has an increased risk for lung cancer susceptibility (GA+GG vs. AA: P=0.022, OR=1.451, 95% CI 1.056-1.994). And the AA variant might be a protective factor in lung cancer. But for the other cancer, like breast cancer and cervical cancer, we did not find any important association in different Aurora-A rs1047972 genotype carriers (Table 3).

#### Association between Aurora-A rs1047972 polymorphism and cancer risk in subgroup analysis by source of control (SC) and genotype methods (GM)

In a stratified analysis by SC, we found an increased risk of ***cancer risk in***PB (population-based study) group (GA+GG vs. AA: P=0.008, OR=1.274, 95% CI 1.064-1.525). Our results indicated that GA / GG genotype carriers have a higher risk of cancer compared to that of AA carriers and the AA variant might be a protective factor for cancer susceptibility in PB group. However, we did not find any association between Aurora-A V57I variant and cancer risk in HB (hospital-based study) group (Table 3).

In a stratified analysis by GM, we observed a significant association between Aurora-A rs1047972 variant and increased risk of cancer in MALDI-TOF group (GA+GG vs. AA: P=0.012, OR=1.436, 95% CI 1.082-1.906). Our results indicated that GA/GG genotype carriers have a higher risk of cancer compared to that of AA carriers (Table 3).

### Sensitivity analysis

In order to evaluate the stability of the analysis results, we conducted a sensitivity analysis by removing each individual research from the analysis at a time. It showed that the changes of each genetic contrast model results were not obvious, suggesting that the results of meta-analysis were stable and reliable.

### Publication bias

**Figure 3:**
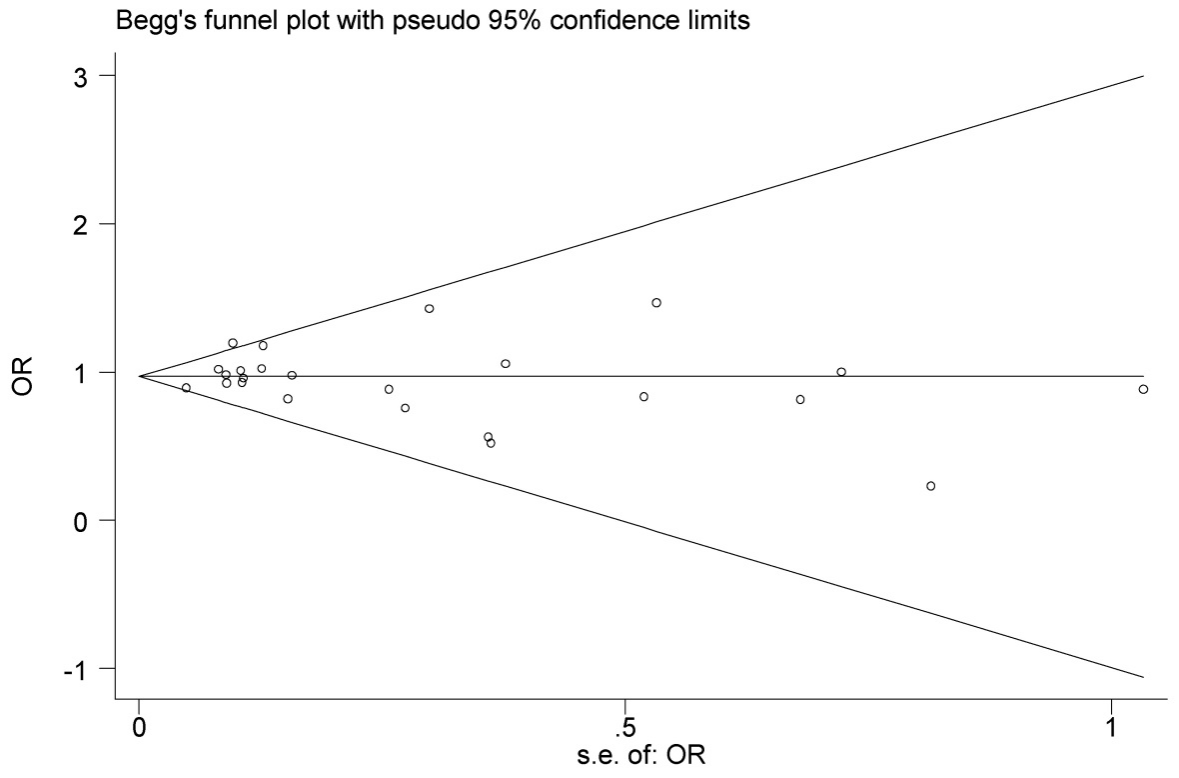
Begg's funnel plot with pseudo 95% confidence limits for studies of the association between cancer risk and Aurora-A V57I variant (the dominant model).

We assessed the publication bias by Begg's funnel plots and Egger's test (dominant model: Egger's test P= 0.952), and the results suggesting that there was no publication bias for the association between Aurora-A rs1047972 gene polymorphism and cancers risk in included studies (Figure 3).

### Trial Sequential Analysis results

**Figure 4:**
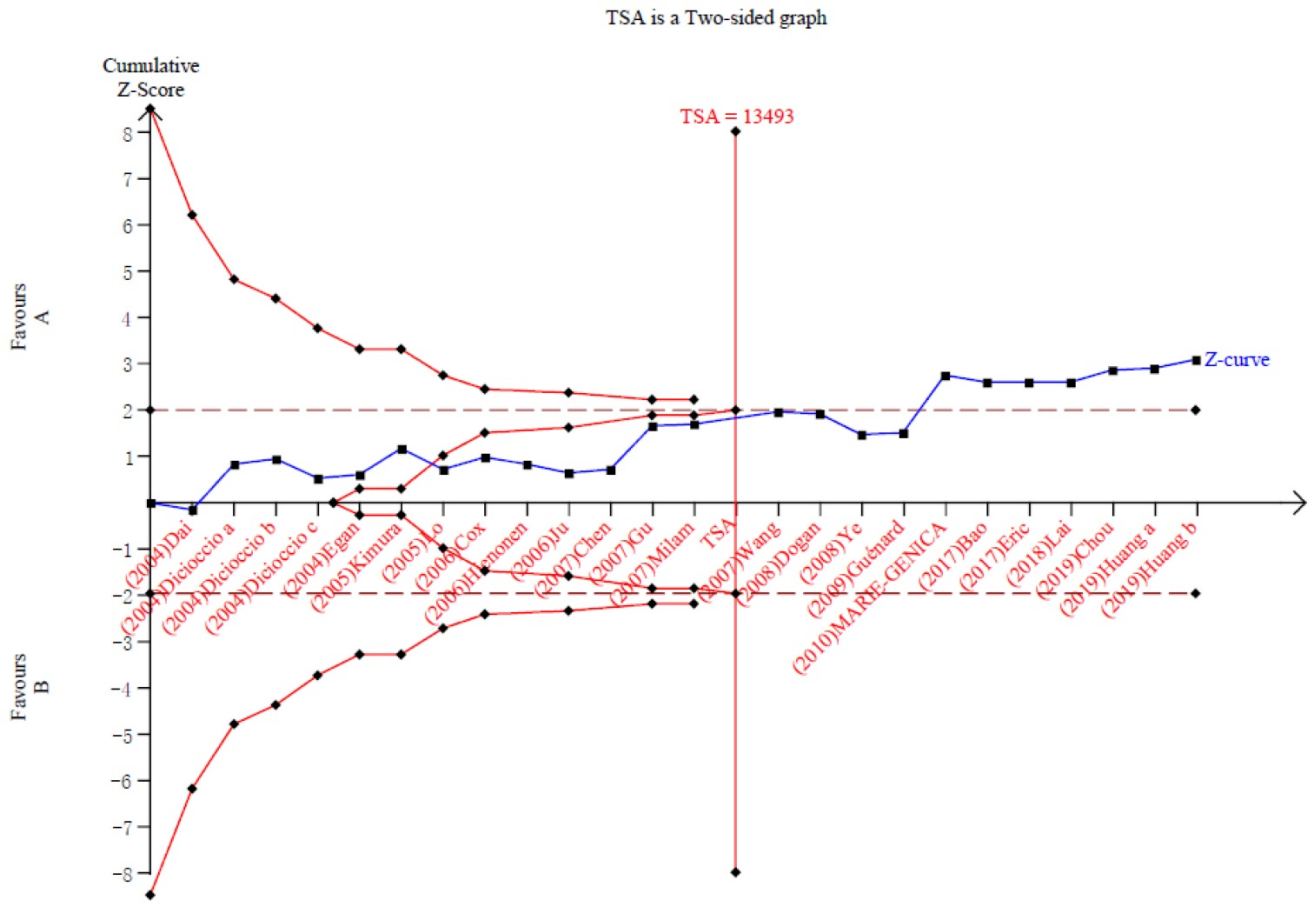
Trial sequential analysis of the association between Aurora-A V57I variant and the risk of cancer. The required information size was calculated based on a two side α= 5%, β = 15% (power 80%), and a relative risk reduction of 20%.

We implemented TSA to reduce the risk of type I error by keeping the overall 5% risk of a type I error and 20% risk of a type II error (power of 80%) to evaluate the RIS. As it showed, the sample size of the 18th study had crossed the TSA boundary (Figure 4). The positive conclusion was obtained in advance, which was consistent with the above meta-analysis results. And the sample size had reached the required information size (13,493 cases) to obtain a positive conclusion. Therefore, it can be thought that AA carriers have a lower risk of cancers than GG carriers, and the evidence is reliable. The actual result was shown in Figure 4.

## Discussion

In the past 20 years, a large amount of epidemiologic researches were centered on correlation between Aurora-A rs1047972 gene polymorphism and cancers risk, but the conclusions are still controversial. Based on it, this meta-analysis was performed to investigate whether the Aurora-A rs1047972 gene polymorphism was correlated with the susceptibility of cancer. In this meta-analysis, a total of 24 case-control studies including 14,639 cancer cases and 21,287 normal controls were investigated. Our results demonstrated that a significant association was observed for Aurora-A rs1047972 gene polymorphism in overall population. And the AA genotype carriers have a slightly lower incidence of cancer compared to that of GA/GG carriers and the GA/GG variant might be a risk factor for cancer susceptibility in overall populations.

In a stratified analysis by population, we found a significant association between Aurora-A rs1047972 variant and the susceptibility of the cancer in Caucasian population. Our results indicated that GA/GG genotype carriers have a higher risk of cancer compared to that of AA carriers and the AA variant might be a protective factor for cancer susceptibility in Caucasian population. However, we did not find any association between Aurora-A rs1047972 variant and cancer risk in Asian population. In a stratified analysis by cancer type, our results indicated that Aurora-A rs1047972 polymorphism has an increased risk for lung cancer.

Our results were consistent with the study by Gu et al. 7. Because Gu et al. also found that Aurora-A rs1047972 polymorphism has an increased risk for lung cancer. But our results were different from Lai et al. 24 and Bao et al. 26, Lai et al. found the association of Aurora-A rs1047972 polymorphism with an increased risk of gastric cancer susceptibility in Malaysians, and gastric cancer incidences among Malaysians have significantly association among younger age group (<50 years). Bao et al. found AA carriers of the Aurora-A rs1047972 polymorphism were significantly associated with decreased susceptibility to HBV-related hepatocellular carcinoma when compared with non-carriers. However, we did not find any significantly association in gastric cancer and hepatocellular carcinoma susceptibility. In addition, Chen et al. 23 found that the Aurora-A rs1047972 polymorphism was associated with protection from colorectal cancer susceptibility. Why the studies above have the different results, the possible reasons might illustrate as below: First, cancer is a complex disease, and various environmental factors might have an influence on the susceptibility of different cancers. During the occurrence and development of malignant tumors, mutations of various tumor suppressor genes and activation of oncogenes are important starting and promoting factors, and they run through the entire process of tumor progression. Second, the difference of ethnic (Asians and Caucasians) in the Aurora-A rs1047972 gene frequencies might also lead to these different results. Third, the other gene polymorphisms might have an effect on the cancer susceptibility, not only the Aurora-A rs1047972 polymorphism. In addition, the increase of various cytokines, chemokines and inflammatory mediators can also promote angiogenesis, cause suppression of local immunity and inhibit apoptosis, and can increase the risk of tumor metastasis.

We also carried out subgroup analysis by SC and GM. In a stratified analysis by SC, we found significant association between Aurora-A rs1047972 polymorphism and increased risk of PB group. In a stratified analysis by GM, we observed significant association between Aurora-A rs1047972 polymorphism and increased risk of MALDI-TOF group. Our results indicated that different genotype methods might have an important affect in different studies.

A previous meta-analysis conducted by Tang et al. 30 included 14 case-control studies. They found Aurora-A rs1047972 polymorphism was a protective factor in Caucasians. However, based on more studies, we found Aurora-A rs1047972 polymorphism was a risk factor in Caucasians. There are some reasons for the different results between Tang et al. and our study: First, the number of patients included in Tang's study was relatively small, and Tang's study was published in 2014, there were many case-control studies in the past five years been published. With more included studies and larger sample sizes, our findings and our results were more accurate and reliable. Second, compared with Tang's study, we also analyzed the relationship between more types of cancer risk and genetic polymorphisms such as cervical cancer, esophageal cancer and hepatocellular carcinoma, which could provide more clues and guidance for clinically practical work. In addition, huge heterogeneity was observed in Tang's study (I^2^ = 55.4%) but not in our meta-analysis (I^2^ = 0%), so this might distort their results.

It is very important for us to pay more attention to the heterogeneity, because huge heterogeneity might have the bad influence on our results of the present meta-analysis and neglect the heterogeneity might lead to mistakes. In the present meta-analysis, the contrast model of Aurora-A rs1047972 polymorphism did not have significant heterogeneity. Therefore, it can be considered the results we performed were reliable in a way. In addition, we carried out the subgroup analysis of other related factors in order to study the relationship between more factors and cancer susceptibility.

Moreover, our meta-analysis has some advantages. Firstly, we have carried out TSA to reduce the article risk of type I error to promote scientific preciseness. Secondly, we have revealed a lot of factors which exert influence on the association between Aurora-A rs1047972 polymorphism and the susceptibility to cancer. Thirdly, strictly based on the search strategy, articles included in our meta-analysis are more than 20 studies which may enhance the authenticity and reliability of the analysis. Fourthly, the subgroup analysis is sufficient and reveals the difference among diverse populations. In addition, Egger's tests and funnel plot were used to find potential publication bias. No significant publication bias was found.

Nevertheless, there are still some limitations which cannot be avoided in our present meta-analysis. First, some unpublished studies might not be included. Second, we did not include studies in other races such as Africa, so it cannot fully reflect the influence of different races and regions. Third, this study did not reveal gene environment and gene-gene interactions, due to the original information of the included studies was not sufficient. Fourth, this study failed to include an independent cohort study into our meta-analysis; therefore, more cohort studies should be performed to refute the association between the risk of cancer and Aurora-A rs1047972 polymorphism in the future. Despite the above limitations, this study ensures the reliability of the results by setting a careful research plan to minimize the bias.

## Conclusion

In summary, the findings from this systematic review and meta-analysis indicate that the susceptibility of cancer was associated with Aurora-A rs1047972 polymorphism, especially, in Caucasians. And the AA genotype carriers have a slightly lower incidence of cancer compared to that of GA/GG carriers and the GA/GG variant might be a risk factor for cancer susceptibility. Our study has certain guiding significance for Aurora-A rs1047972 polymorphism and cancer research, and contributes to the development of ARUKA inhibitors. Of course, due to the research we included in this meta-analysis was the only case-control study, more prospective study should be performed to refute the association between the risk of cancer and Aurora-A rs1047972 polymorphism in the future.

## Figures and Tables

**Table 1:** Main characters of studies included in this meta-analysis

First author	Year	Country	Ethnicity	Cancer type	SC	Case(n)	Control(n)	GM	NOS score
Egan	2004	USA	Caucasian	Breast cancer	PB	905	788	PCR	8
Dicioccio a	2004	UK	Caucasian	Ovarian cancer	PB	750	843	TaqMan	7
Dicioccio b	2004	USA	Caucasian	Ovarian cancer	PB	323	427	TaqMan	7
Dicioccio c	2004	Denmark	Caucasian	Ovarian cancer	PB	432	1112	TaqMan	7
Dai	2004	China	Asian	Breast cancer	PB	1102	1186	TaqMan	8
Lo	2005	China	Asian	Breast cancer	HB	704	1950	TaqMan	8
Kimura	2005	Japan	Asian	Esophageal cancer	HB	197	146	PCR	7
Hienonen	2006	Finland	Caucasian	Colorectal cancer	HB	125	94	PCR	8
Cox	2006	USA	Caucasian	Breast cancer	PB	1240	1724	TaqMan	9
Ju	2006	Korea	Asian	Gastric cancer	HB	501	427	PCR	8
Gu	2007	USA	Caucasian	Lung cancer	HB	1098	1027	TaqMan	9
Chen	2007	USA	Caucasian	Colorectal cancer	HB	60	65	PCR	6
Milam	2007	USA	Caucasian	Cervical cancer	HB	140	188	TaqMan	7
Wang	2007	USA	Caucasian	Lung cancer	HB	1263	1154	TaqMan	7
Ye	2008	USA	Caucasian	Bladder cancer	HB	604	593	TaqMan	9
Dogan	2008	Turkey	Caucasian	Lung cancer	HB	102	102	PCR	8
Guénard	2009	Canada	Caucasian	Breast cancer	HB	96	96	PCR	7
MARIE-GENICA	2010	German	Caucasian	Breast cancer	PB	3139	5469	MALDI-TOF	9
Lai	2018	Malaysia	Asian	Gastric cancer	PB	41	1110	PCR-RFLP	6
Eric	2017	Malaysia	Asian	Breast cancer	HB	71	260	PCR-RFLP	6
Bao	2017	China	Asian	Hepatocellular carcinoma	HB	348	359	PCR-RFLP	9
Chou	2019	China	Asian	Oral cancer	HB	876	1200	PCR	8
Huang a	2019	China	Asian	Urothelial Cell Carcinoma	HB	431	862	TaqMan	7
Huang b	2019	China	Asian	Oral cancer	HB	91	105	PCR-RFLP	8

Abbreviations: PCR, polymerase chain reaction; RFLP, restriction fragment length polymorphism; SC, source of control; GM, genotype methods; HB, hospital-based study; PB, population-based study; MALDI-TOF: Matrix-Assisted Laser Desorption/Ionization Time of Flight Mass spectrometry; NA, not available.

**Table 2:** Meta-analysis results

Study	Contrast model	OR (95%Cl)	P	Test for heterogeneity		Publication bias (Egger's test)		Analysis model
				I^2^ (%)	P	t	P	
Overall	AA vs.GG	0.814(0.710,0.934)	0.003	0.00%	0.535	1.49	0.150	F
	GA vs.AA	1.079(0.977,1.191)	0.134	13.20%	0.279	-0.02	0.987	F
	AA+GA vs.GG	0.960(0.912,1.010)	0.118	3.50%	0.413	-0.06	0.952	F
	GG+GA vs.AA	1.106(1.005,1.218)	0.039	18.30%	0.210	-0.19	0.854	F
Asian	AA vs.GG	0.872(0.662,1.150)	0.333	15.40%	0.301			F
	GA vs.AA	0.986(0.868,1.121)	0.833	22.80%	0.233			F
	AA+GA vs.GG	0.906(0.736,1.116)	0.353	39.90%	0.092			R
	GG+GA vs.AA	1.017(0.898,1.151)	0.793	30.30%	0.166			F
Caucasian	AA vs.GG	0.797(0.681,0.933)	0.005	0.00%	0.636			F
	GA vs.AA	1.234(1.054,1.445)	0.009	0.00%	0.666			F
	AA+GA vs.GG	0.952(0.900,1.008)	0.090	0.00%	0.819			F
	GG+GA vs.AA	1.254(1.077,1.461)	0.004	0.00%	0.609			F

Abbreviations: R: random-effect model; F: fixed-effect model; OR: odds ratio; CI: confidence interval.

**Table 3:** Subgroup of Meta-analysis results

Study	Subgroup	Contrast model	OR (95%Cl)	P	Test for heterogeneity		Analysis model
					I^2^ (%)	P	
Cancer type	Lung cancer	GG+GA vs.AA	1.451(1.056,1.994)	0.022	0.00%	0.706	F
	Breast cancer	GG+GA vs.AA	1.153(0.962,1.382)	0.123	38.90%	0.132	F
	Ovarian cancer	GG+GA vs.AA	1.139(0.768,1.687)	0.518	0.00%	0.487	F
	Esophageal cancer	GG+GA vs.AA	1.123(0.716,1.763)	0.613	NA	NA	F
	Colorectal cancer	GG+GA vs.AA	0.713(0.271,1.878)	0.494	0.00%	0.389	F
	Gastric cancer	GG+GA vs.AA	1.173(0.704,1.955)	0.541	44.80%	0.178	F
	Oral cancer	GG+GA vs.AA	1.282(0.750,2.190)	0.364	63.80%	0.096	R
	Cervical cancer	GG+GA vs.AA	1.248(0.293,5.311)	0.765	NA	NA	F
	Bladder cancer	GG+GA vs.AA	0.593(0.296,1.188)	0.141	NA	NA	F
	Hepatocellular carcinoma	GG+GA vs.AA	0.786(0.560,1.103)	0.164	NA	NA	F
	Urothelial Cell Carcinoma	GG+GA vs.AA	1.132(0.868,1.478)	0.360	NA	NA	F
SC	PB	GG+GA vs.AA	1.274(1.064,1.525)	0.008	0.00%	0.800	F
	HB	GG+GA vs.AA	1.044(0.932,1.170)	0.454	29.10%	0.132	F
GM	PCR	GG+GA vs.AA	1.053(0.894,1.241)	0.535	0.00%	0.894	F
	TaqMan	GG+GA vs.AA	1.137(0.973,1.327)	0.105	0.30%	0.437	F
	MALDI-TOF	GG+GA vs.AA	1.436(1.082,1.906)	0.012	NA	NA	F
	PCR-RFLP	GG+GA vs.AA	1.041(0.632,1.714)	0.875	70.00%	0.019	F

Abbreviations: R: random-effect model; F: fixed-effect model; OR: odds ratio; CI: confidence interval; SC, source of control; GM, genotype methods; HB, hospital-based study; PB, population-based study; NA, not available.

## Abbreviations

OR: odds ratio
CI: confidence interval
HWE: Hardy-Weinberg equilibrium
AURKA: Aurora-A
SNP: single nucleotide polymorphism
the NOS scale: the Newcastle-Ottawa Scale
R: random-effect model
F: fixed-effect model
SC: source of control
GM: genotype methods
PCR: polymerase chain reaction
RFLP: restriction fragment length polymorphism
HB: hospital-based study
PB: population-based study
